# Transforming growth factor-β1 gene promoter -509C/T polymorphism in association with expression affects colorectal cancer development and depends on gender

**DOI:** 10.1371/journal.pone.0201775

**Published:** 2018-08-02

**Authors:** Spaska Stanilova, Noyko Stanilov, Alexander Julianov, Irena Manolova, Lyuba Miteva

**Affiliations:** 1 Department of Molecular Biology, Immunology and Medical Genetics, Medical Faculty, Trakia University, Stara Zagora, Bulgaria; 2 Breast Oncoplastic Unit, University College London Hospital, London, United Kingdom; 3 Trakia Hospital, Stara Zagora, Bulgaria; 4 Department of Surgery, Medical Faculty, Trakia University, Stara Zagora, Bulgaria; University of South Alabama Mitchell Cancer Institute, UNITED STATES

## Abstract

It is widely known that sporadic colorectal cancer (CRC) is age-related diseases with higher incidence rate among men. Transforming growth factor-β1 (TGF-β1) is a major immune regulatory cytokine with a great impact and dual role in gastrointestinal carcinogenesis. In this context, the aim of the study was to explore the role of circulating TGF-β1 and the -509C/T functional promoter polymorphism (rs1800469) within the TGF-β1 gene (*TGFB1*) in the susceptibility, progression, and prognosis of CRC among Bulgarian male and female patients. Patients with sporadic CRC and healthy controls were genotyped by polymerase-chain reaction–restriction fragment length polymorphism. Serum TGF-β1 levels before and after curative surgery were determined by ELISA. Total RNA was extracted from paired tumor, normal mucosa and distant metastasis samples and was used for quantitative detection of *TGFB1* mRNA by TaqMan qPCR.We observed that TGF-β1 serum levels depend on the -509C/T genotype in combination with gender. TGF-β1 serum levels in CRC patients were decreased compared to controls, but statistical significance was reached only for men. In the stratified analysis by gender and genotype, a significant association was found for the CC genotype. Overall, our results indicate that the -509C allele increased the cancer risk, particularly for advanced stages (OR = 1.477; p = 0.029). The results from the relative mRNA quantification showed a significant upregulation of *TGFB1* in distant metastases compared to primary tumor tissues and higher *TGFB1* mRNA levels in men (RQ = 4.959; p = 0.022). In conclusion, we present data that diminished circulating TGF-β1 due to the CC genotype could be a possible risk factor for tumor susceptibility and progression. This association is more pronounced in males than in females. Colorectal cancer tissue expression of *TGFB1* gene mRNA correlates with tumor progression and metastasis.

## Introduction

Transforming growth factor-β1 (TGF-β1) is a major immune regulatory cytokine with a great impact on gastrointestinal tumorigenesis. TGF-β1 belongs to a large ancient family of multifunctional proteins that are secreted by a variety of cell types and act as signal molecules in controlling a great number of biological processes, including cell differentiation, adult tissue homeostasis, wound healing and immune regulation [1,2]. TGF-β1 is produced by both immune and non-immune cells. In epithelial cells, the cellular response to TGF-β1 includes transmembrane serine/threonine kinase receptors (TGF-βRI and TGF-βRII), which upon ligand binding recruit and phosphorylate a specific subset of Smad proteins in a cascade manner [3]. This canonical signalling cascade, known as the Smad-dependent pathway, leads to the transcription of particular genes. Non-canonical signalling pathways can involve other intracellular proteins. TGF-β1 is a cytokine with regulatory activities that orchestrates the differentiation of both T regulatory (Tregs) and T helper 17 (Th17) cells in a concentration-dependent manner–low doses induce Th17 cell differentiation, while high doses inhibit Th17 cell development and promote Tregs [4,5]. One of the mechanisms by which TGF-β1 is able to maintain tolerance is to support survival and regulatory functions through the upregulation of Foxp3 expression in peripheral CD4+CD25+ regulatory T cells [6]. Another function of Tregs is to suppress host anti-tumor response and promote tumor development [7].

TGF-β1 signalling is considered to play a dual role in carcinogenesis [8]. Both tumor suppressive and pro-oncogenic TGF-β1 activities have been published. In normal and premalignant epithelial cells, TGF-β1 generally suppresses tumor progression, but TGF-β pathway dysregulation under certain conditions leads to extensive signal reprogramming, which allows cancer cells to survive and successfully spread in other tissues [9,10]. As the role of TGF-β1 in tumor development includes both pro- and anti-carcinogenic effects, the exact conditions required for its proper activity are still poorly understood. The question of how TGF-β1 achieves its distinct effects in tumor development remains unanswered. One possibility is the distinct role of systemic and local quantities of endogenous TGF-β1 proteins, which depends on many factors, including promoter polymorphisms in the TGF-β1 gene (*TGFB1*).

Recently, a genome-wide association (GWA) study identified low-risk variant loci on chromosomes 8q23.3 (EIF3H), 8q24, 10p14, 11q23, 15q13, 16q22, 18q21 (SMAD7), 19q13 and 20p12, which contribute to the genetic risk of colorectal cancer susceptibility [11]. The TGF-β1 gene is located on chromosome 19 (q13.1–13.3) and encodes a 25-kDa multifunctional homodimer protein consisting of 112 amino acids. Several polymorphisms have been described in the coding and regulatory sequences of the TGF-β1 gene, including a promoter polymorphism involving a C-to-T transition (-509C/T) [12]. A study of twins estimated that the -509C/T polymorphism is significantly associated with the plasma concentration of both acid-activated latent and active TGF-β1, demonstrating 8.2% of the additive genetic variance to its concentration [13]. The -509C/T single nucleotide polymorphism (SNP) is located within a YY1 consensus binding site, and the -509C allele has been associated with selective AP1 recruitment to the *TGFB1* promoter that can downregulate *TGFB1* transcription [14]. Low T-cell proliferation, which correlates with higher plasma TGF-β1 concentrations in subjects with the T allele, has been discovered in patients with allergies [15].

A number of studies have investigated the role of the *TGFB1* promoter polymorphism -509C/T in susceptibility to CRC. The meta-analysis by Fang and colleagues showed that the *TGFB1* -509C allele is a risk factor for developing colorectal cancer in Asians, though no significant association was found among Europeans [16]. In a meta-analysis of 55 case-control studies on the association of the -509C/T SNP with cancer risk, including 12 studies on colorectal cancer, the T allele in this polymorphism was associated with a decreased risk of colorectal cancer (TT vs. CT+CC: OR = 0.85, 95% CI = 0.76–0.95), especially for Caucasians in population-based studies [17]. However, some studies have failed to find a relationship between the *TGFB1* -509C/T polymorphism and colorectal cancer risk in a Chinese population [18,19], and therefore, whether there is an association between this polymorphism and colorectal cancer risk is unclear and requires further studies.

Recently developed gene-sequencing technologies have revealed clear disparities in the distribution of genetic polymorphisms in populations stratified by ethnicity [20]. The ethnic background also plays a significant role in affecting the association between supposed genetic markers and cancer risk. Jing and colleagues in a systematic review paper investigated 82 SNPs in association with six different types of cancers. They have demonstrated that the effect of the studied alleles on cancer risk showed the significant heterogeneity among populations of European, Asian and African origin [21].

Bearing in mind the above results, we aimed to investigate the association between the *TGFB1* -509C/T polymorphism and the susceptibility to colorectal cancer in a Bulgarian population. We also evaluated the relationship between this polymorphism and TGF-β1 serum levels in healthy and patient groups as well as *TGFB1* expression in tumor tissues.

## Materials and methods

### Ethics statement

The protocol for this population-based case-control study was approved by the University Hospital Ethics Committee, and written informed consent was obtained from the patients and healthy controls.

### Study subjects

The study recruited 185 newly diagnosed and histologically confirmed colorectal cancer patients who were operated on at the University Hospital and Trakia Hospital, Stara Zagora, Bulgaria. The 210 healthy controls (HC) were selected from the same cohort as the corresponding patients. The following exclusion criteria were used for the patients and controls: previous diagnosis of autoimmune or inflammatory bowel disease or individual history of cancer and family history of any known hereditary cancer syndromes. Patients did not receive chemotherapy or radiation therapy before surgery. Pathological tumor staging and grading was performed according to the TNM classification. Cases with stage I and II were collectively noted as cases with early CRC, and cases with stage III and IV—as advanced CRC. There is no significant difference between male and female CRC patients according to the clinical characteristics, including stage, T- N- and M- status, location, differential degree of CRC. The slightly higher prevalence of the rectal cancer was detected in male patients than female (χ^2^ = 2.859; p = 0.091) without reaching the statistical significance. The patients consisted of 116 (62.7%) males and 69 (37.3%) females from 55 to 78 years old with a mean age of 65.34 years, without significant differences in age between males and females (p = 0.733; t-test). The healthy controls were consecutively recruited from participants in a health check-up programme during the study period. The circulatory TGF-β1 levels in patients were compared to matched controls over the age of 50 yrs with similar gender distribution (χ^2^ = 0.055; p = 0.814). The demographic and clinical characteristics of the patients and control subjects are summarized in Table 1.

**Table 1:** The demographic characteristics of the CRC patients and control subjects.

Characteristic	Total	Female	Male
**Gender, n (%)**			
Controls	210 (100)	108 (51.4)	102 (48.6)
CRC patients	185 (100)	69 (37.3)	116 (62.7)
**mean Age ±s.d. (years)**			
Total Controls	53.1±12	50.6±10.4	55.8±13
CRC patients	65.3±10.4	65.7±10.4	65.1±10.5
**<50 yrs, n (%)**			
Controls	100 (47.6)	64 (64)	36 (36)
CRC patients	14 (7.6)	3 (21.4)	11 (78.6)
**>50 yrs, n (%)**			
Matched Controls*	110 (52.4)	44 (40)	66 (60)
CRC patients	171 (92.4)	66 (38.6)	105 (61.4)
**TNM pathological stage**			
I stage, n (%) II stage, n (%) III stage, n (%) IV stage, n (%)	22 (11.9) 66 (35.7) 58 (31.3) 39 (21.1)	8 (11.6) 26(37.7) 25 (36.2) 10 (14.5)	14 (12.1) 40 (34.5) 33 (28.4) 29 (25)
**Early CRC, n (%)**	88 (47.6)	34 (49.3)	54 (46.6)
**Advanced CRC, n (%)**	97 (52.4)	35 (50.7)	62 (53.4)
**Local tumor invasion**			
T1-T2	30 (16.2)	15 (21.7)	15 (12.9)
T3-T4	155 (83.8)	54 (78.3)	101 (87.1)
**Lymph nodes involvement**			
N0	105 (56.8)	37 (53.6)	68 (58.6)
N1-2	80 (43.2)	32 (46.4)	48 (41.4)
**Distant metastasis**			
M0	144 (77.8)	58 (84.1)	86 (74.1)
M1-2	41 (22.2)	11 (15.9)	30 (25.9)
**Tumor location**			
Colon, n (%)	87 (47)	38 (55.1)	49 (42.2)
Rectum, n (%)	98 (53)	31 (44.9)	67 (57.8)
**Differentiation degree**			
Well, n (%)	41 (22.2)	16 (23.2)	25 (21.6)
Moderate, n (%)	102 (55.1)	35 (50.7)	67 (57.8)
Poor, n (%)	42 (22.7)	18 (26.1)	24 (20.7)

* Matched Controls were used for analyses of serum TGF-β1 levels

### Blood samples and DNA extraction

Blood samples were collected for serum separation and DNA isolation in appropriate blood collectors from all included subjects. Serum samples were frozen in small aliquots at −70°C until analysis. Ten days after the surgical removal of the tumors, blood samples were taken from patients to analyse post-operative cytokine levels. Serum samples from patients and control subjects were analysed together in the same analytic batch. Genomic DNA was extracted from 200 μl of venous blood using genomic DNA purification kits (GeneJET Genomic DNA Purification Kit, Thermo Fisher Scientific) and stored at -80°C until use. The purification procedure was performed according to the manufacturer’s protocol. The concentration and purity of the DNA samples were measured spectrophotometrically at 260 nm/280 nm using a GeneQuant 1300 spectrophotometer (GE Healthcare Life Sciences, Switzerland).

### Genotyping for the *TGFB1* gene -509C/T polymorphism

Genotyping the -509C/T polymorphism in the *TGFB1* promoter (rs1800469) was performed by polymerase chain reaction–restriction fragment length polymorphism (PCR-RFLP) assay. Amplification of the 153 bp fragment was performed with the 5’–CAGTAAATGTATGGG GTCGCA G–3’ forward primer and 5’–GGTGTCAGTGGGAGGAGGG–3’ reverse primer at the follow cycling parameters: initial incubation step of 3 min at 95°C; 30 cycles of 45 seconds at 94°C, 45 seconds at 63.3°C, and 45 seconds at 72°C; and a final extension step of 7 min at 72°C to complete the reaction. The amplified products (10 μl) were digested using 10 U Eco81I (Thermo Fisher Scientific) per reaction for 12 hours at 37°C. The -509C allele yields two fragments at 115 bp and 38 bp.

To verify the genotyping analysis, a second amplification reaction was performed with another set of primers, the 5’-CGGACACCCAGTGATGGG-3’ and in reverse direction: 5’- CCTCCTGGCGGCCAAGCGC-3’. The samples were denatured at 94°C for 3 min, followed by 30 cycles of denaturation at 94°C for 45 sec, annealing at 60°C for 45 sec, and extension at 72°C for 45 sec, and a final extension step at 72°C for 10 min. The resulting 530 bp PCR products were digested for 12 h at 37°C with the 10U Eco81I. The -509C allele yields two fragments at 273 bp and 257 bp, which were detected on a 2% agarose gel and visualized with ethidium bromide. The PCR was performed in a GeneAmp PCR System 9700 (Applied Biosystems, Foster City, CA, USA). The PCR reagents were provided by Thermo Fisher Scientific (USA). The primers were supplied by Metabion GmbH (Germany). For quality control, approximately 10% of random selected samples, including both patients and controls, were reanalysed using a heterozygous control template without finding any discrepancies in the PCR runs.

### Tissue specimens and qPCR detection of *TGFB1* expression

Paired tissue samples from the tumoral and non-tumoral mucosa and distant metastases were taken from CRC patients. The tissue samples (30 mg) were harvested immediately after resection and were used for RNA isolation. Total RNA was isolated using a GeneJet RNA purification kit (Thermo Fisher Scientific) according to the manufacturer’s instructions. The total RNA was quantified spectrophotometrically (GeneQuant 1300 spectrophotometer, GE Healthcare Life Sciences, Switzerland). Synthesis of cDNA was performed with random hexamer primers using a RevertAid First Strand cDNA Synthesis Kit (Thermo Fisher Scientific). Reverse-transcription PCR was performed on a GeneAmp PCR System 9700 (Applied Biosystems, Foster City, CA, USA).

Quantitative real-time polymerase chain reaction (qPCR) was performed by using TaqMan gene expression assays on a 7500 Real-Time PCR System (Applied Biosystems, Foster City, CA, USA) and the data were collected with Sequence Detection System (SDS) software, version 1.3.1. The predesigned inventoried TaqMan gene expression assays for *TGFB1* (Hs00998133_m1) and for the endogenous controls/reference genes *GAPDH* (Hs02758991_g1) and eukaryotic 18S ribosomal RNA (Hs99999901_s1) were purchased from Thermo Fisher Scientific. The procedure is described in detail in our previous study [7].

### Quantification of serum TGF-β1 levels

The serum concentrations of latent acid-activated TGF-β1 protein were determined by the quantitative sandwich Enzyme-linked immunosorbent assay (ELISA) method according to the manufacturer’s instructions (Quantikine ELISA Kits, R&D systems, Minneapolis, MN, USA). Latent serum TGF-β1 was activated by acid activation by 1N HCl and neutralization by 1.2N NaOH/0.5M HEPES, following the manufacturer instructions. The activated samples were stored at 4°C for less than 16 hours. Serum samples from the patients and controls were analysed together in the same analytic batch. The results were calculated by reference to the standard curve and expressed as nanograms per ml (ng/ml). The minimum detectable TGF-β1 levels ranged from 1.7–15.4 pg/ml. The standards curve was constructed by using provided by manufacturer standards within the range from 0 pg/ml to 2000 pg/ml.

### Statistical analysis

The genotype and allele frequencies among the patients and controls, as well as the test for deviation from the Hardy-Weinberg equilibrium, were analysed by the χ^2^ test. The StatPages.net website (http://statpages.org/index.html) was used to estimate the odds ratios (ORs) with 95% confidence intervals (95%CI) for disease susceptibility related to the *TGFB1* -509C/T polymorphism. The non-parametric Mann-Whitney U test was used to compare the serum concentration of TGF-β1 among the patients and controls and between the different -509C/T genotypes. The results are presented as the mean ± standard error (SE). Relative quantitative analysis of gene expression was performed by the comparative ddCt method, and the results are presented as an n-fold mean difference (RQ-relative quantity) in the *TGFB1* mRNA levels calibrated to the non-tumoral mucosa after normalization to the geometric mean of the two reference genes. The data were analysed using the StatSoft software. In all cases, two-tailed p-values less than 0.05 were considered significant.

## Results

### TGF-β1 serum levels depend on the *TGFB1* -509C/T polymorphism as well as age and gender in healthy controls

**Fig 1:**
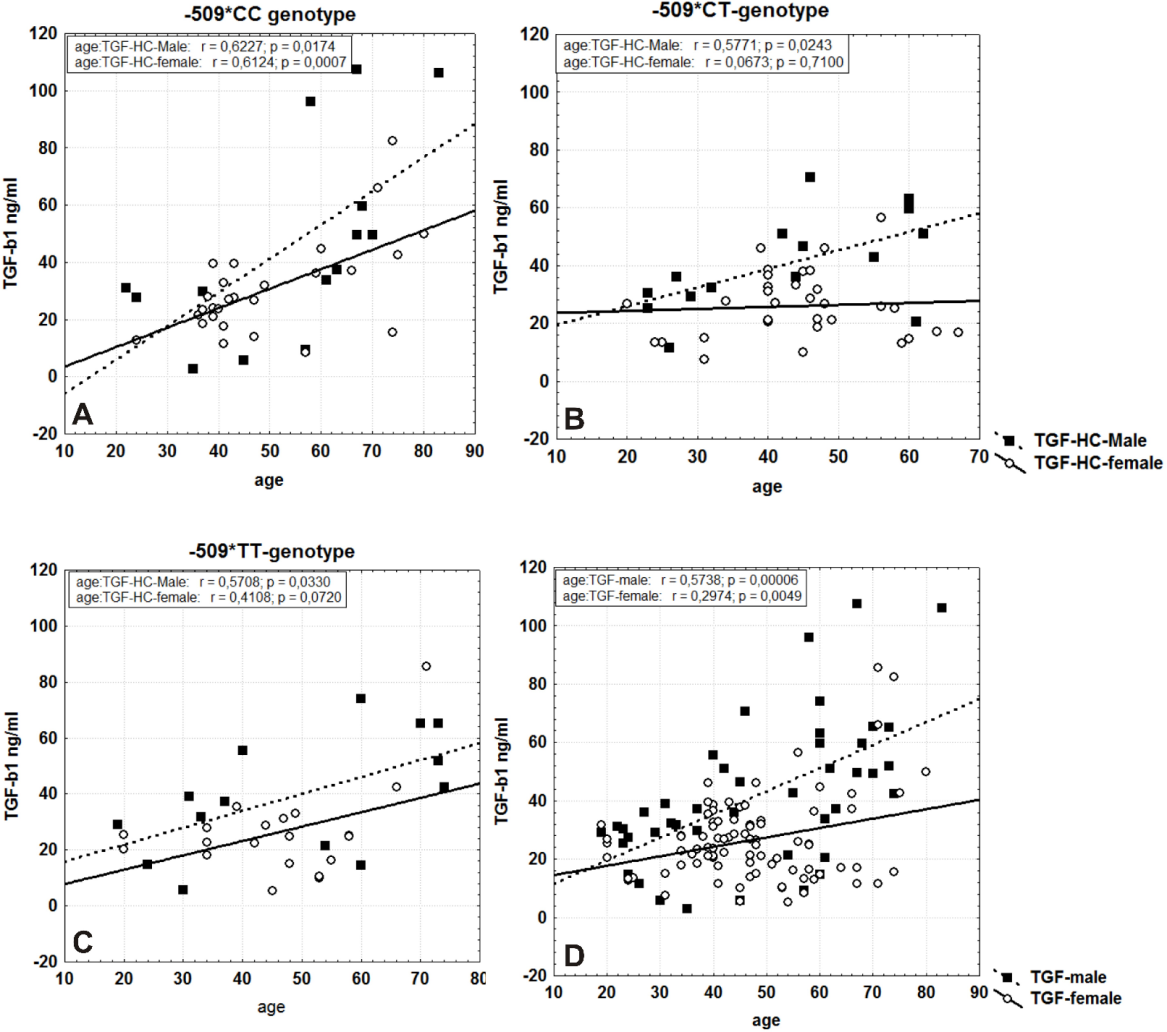
Gender-related correlation between the *TGFB1*-509 polymorphism and serum TGF-β1 levels in healthy controls. (A) CC genotype. (B) CT genotype. (C) TT genotype. (D) the total group. The individual data from females are presented as empty circles, and those from males are presented as filled squares.

The results for the TGF-β1 serum levels HCs depend on the *TGFB1*-509C/T genotype, age and gender (Fig 1).

**Fig 2:**
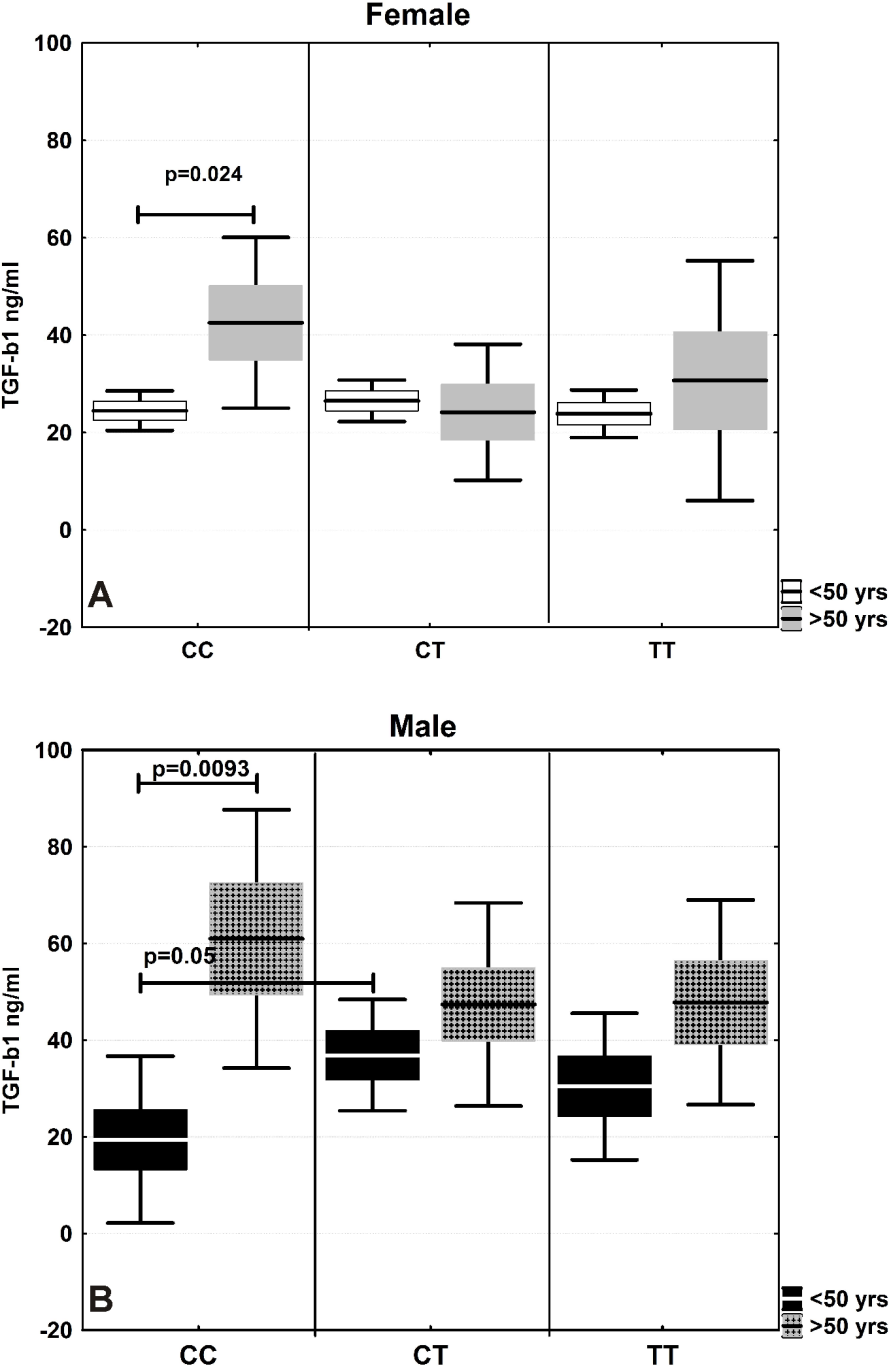
TGF-β1 serum levels in controls, aged under and over 50 in relation to *TGFB1*-509 polymorphism. (A) Females over 50 (>50) compared to females under 50 (<50). (B) Males over 50 (>50) compared to males under 50 (<50). The results are presented as the mean value ±SE (box) with a ±0.95 confidential interval (whisker).The statistical significance of the U-test is shown as the p-value.

In males, a strong to moderate positive correlation was observed between age and serum TGF-β1 levels in all three genotypic groups. In women, this significant relationship was detected in the carriers of the CC genotype, although the TT genotype showed a similar trend, without significance. Males generally have higher serum TGF-β1 levels than females (41.84 ± 3.8 vs. 26.54 ± 1.6 ng/ml, respectively; p = 0.028), and this association increases with age (Fig 1D). Moreover, the differences were also dependent on the genotype, and they are most pronounced for males and females with at least one C allele in the genotype (Fig 1A). When healthy males and females were subdivided into two groups, under and over 50 years, we calculated a significant difference for only the CC genotype in both men (19.43±6.2 vs. 60.95±11.6ng/ml; p = 0.009, U-test) and women (24.49±1.9 vs. 42.54±7.6ng/ml; p = 0.0236, U-test) (Fig 2).

### Association of the *TGFB1*-509C/T polymorphism with a susceptibility to colorectal cancer

The genotype distribution and allele frequencies of the -509C/T SNP in the *TGFB1* gene promoter among colorectal cancer patients and healthy controls are presented in Table 2.

**Table 2:** The genotype distribution and allele frequencies of the -509C/T SNP in the *TGFB1* gene promoter among colorectal cancer patients and healthy controls.

*CRC vs*. *Healthy controls*	n (%)	CC n(%)	CT n(%)	CC+CT n(%)	TT n(%)	C n(%)	T n(%)
**Early CRC**	**88 (100)**	25 (28)	44 (50)	69 (78)	19 (22)	94 (53)	82 (47)
ОR (95% CI), *p* value **vs.** **Healthy controls**		0.937 (0.437–2.011) P = 0.856	1.122 (0.564–2.241) P = 0.725	1.047(0.551–2.001) P = 0.881	1.0 (ref.)	0.956 (0.661–1.382) P = 0.803	1.0 (ref.)
**Advanced CRC**	**97 (100)**	37 (38)	50 (52)	87 (90)	10 (10)	124 (64)	70 (36)
OR (95% CI), *p* value **vs.** **Healthy controls**		**2.635 (1.123–6.304)** **P = 0.015**	**2.423 (1.071–5.600)** **P = 0.021**	**2.509 (1.154–5.584)** **P = 0.011**	1.0 (ref.)	**1.477 (1.025–2.130)** **P = 0.029**	1.0 (ref.)
**CRC**	**185 (100)**	62 (33)	94 (51)	156 (84)	29 (16)	218 (59)	152 (41)
OR (95% CI), *p* value **vs.** **Healthy controls**		1.522 (0.821–2.831) P = 0.153	1.571 (0.882–2.804) P = 0.102	1.551 (0.902–2.674) P = 0.092	1.0 (ref.)	1.196 (0.893–1.603) P = 0.214	1.0 (ref.)
**Healthy controls**	**210 (100)**	66 (31)	97 (46)	163 (78)	47 (22)	229 (55)	191 (45)
OR (95% CI), *p* value ***Advanced vs*. *Early***		**2.812 (1.025–7.834)** **P = 0.025**	***2*.*159 (0*.*840–5*.*627)*** ***P = 0*.*078***	**2.396 (0.978–5.957)** **P = 0.035**	1.0 (ref.)	**1.545 (0.997–2.396)** **P = 0.040**	1.0 (ref.)

Early CRC includes I and II stage by TNM; advanced CRC includes III and IV stage by TNM. OR- odds ratio; 95% CI–confidentail intervals

The genotype distribution for the *TGFB1* -509C/T polymorphism was in agreement with the Hardy-Weinberg equilibrium among patients (χ^2^ = 0.369; p = 0.083) and controls (χ^2^ = 1.046; p = 0.59).

Differences in the *TGFB1* -509C/T genotype distribution between CRC patients and controls were insignificant (χ^2^ = 2.864; p = 0.239). However, a clear tendency for a decrease in the TT genotype was observed in the CRC group (16% vs. 22%). The main differences in the genotype distribution were observed between advanced and early stages of CRC. The presence of the C allele in homo–or heterogeneous genotypes was overrepresented among advanced cancer patients (90%) compared to both early CRC patients (78%; p = 0.035) and HCs (78%; p = 0.011). The genotype distribution of the *TGFB1* -509C/T polymorphism between cases of 1^st^ and 2^nd^ stages of CRC was very similar (χ^2^ = 0.278; p = 0.87) as well as between 3^rd^ and 4^th^ stages (χ^2^ = 0.153; p = 0.926). In addition, the genotype frequencies in 1^st^ and 2^nd^ stages of CRC were close to that in controls. While the CC and CT genotypes were in higher frequency in the 3^rd^ stage (40% and 50%) and in 4^th^ stage (36% and 54%) than controls (31% and 46%, respectively) with borderline significance. The results for allelic frequencies among cases in different stages of CRC were in the same direction.

As shown in Table 2, a significantly increased risk for advanced CRC was found for CC versus TT (OR = 2.635; 95% CI 1.123÷6.304; p = 0.015), CT versus TT (OR = 2.423; 95% CI 1.071÷5.6; p = 0.021), and CC + CT versus TT (OR = 2.509; 95% CI 1.154÷5.584; p = 0.011). Overall, our results indicate that the -509C allele increased the cancer risk, especially for advanced stages (OR = 1.477; 95% CI 1.025÷2.130; p = 0.029), while the observed genotype and allelic frequencies in early CRC were similar to that in healthy controls. Using logistic regression analysis, the CC genotype and C allele appeared as susceptible, while the TT genotype and T allele appear as protective against CRC development and progression to advanced stages.

### TGF-β1 serum levels in colorectal cancer patients

**Fig 3:**
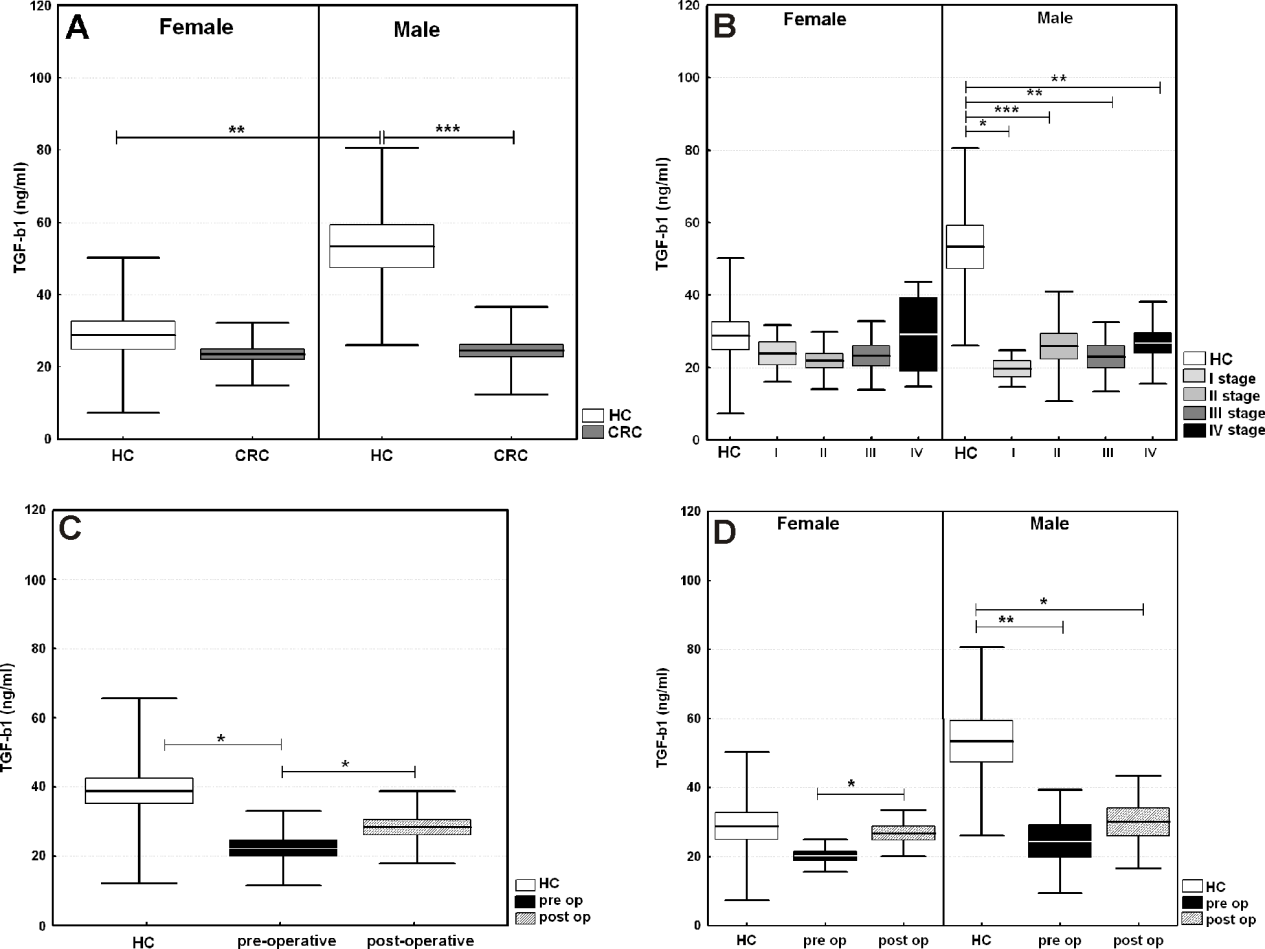
Serum levels of TGF-β1 in CRC patients. (A) Subdivided according to the gender. (B) CRC stage. (C) operative status. (D) operative status in men and women with CRC. The results are presented as the mean value ±SE (box) and ±SD (whisker). The statistical significance of the U-test is shown. * p-value < 0.05, ** p-value < 0.01, and *** p-value < 0.001.

With respect to the observed significant correlation between serum TGF-β1 levels and age in the healthy group and the mean age of CRC patients, the observed circulatory TGF-β1 levels in patients were compared to those of controls over the age of 50 yrs (Fig 3). Serum levels from patients with CRC were decreased compared to those of the controls, and this lower level was more pronounced and significant for males (24.47±1.7 vs. 53.35±5.9 ng/ml; p = 0.000012). In contrast, for female patients, the average value was similar to that of the female controls (23.49±1.4 vs. 28.77±3.9 ng/ml; p = 0.86) (Fig 3A). Obviously (due to cancer development), the serum levels between male and female patients was equal. Moreover, male patients with all stages of CRC had significantly lower levels than the controls. The sex differences in TGF-β1 serum levels in the subgroups by CRC stage are shown in Fig 3B. Once again, in contrast to women, male CRC patients showed lower levels than the controls independent of the CRC stage (Fig 3B). The levels of TGF-β1 increased significantly after surgery (22.20±2.4 vs. 28.36±2.2 ng/ml; p = 0.014) but remain diminished compared to those of controls (Fig 3C). The increase in serum TGF-β1 levels in male patients after surgery did not reach the healthy donor values, in contrast to that in female patients (Fig 3D). Among men, post-operative TGF-β1 levels were significantly lower in CRC patients than in controls (29.6±4.4 vs. 53.4±5.9 ng/ml; p = 0.016).

#### Association of *TGFB1*-509C/T polymorphism with TGF-β1 serum levels in CRC patients

**Fig 4:**
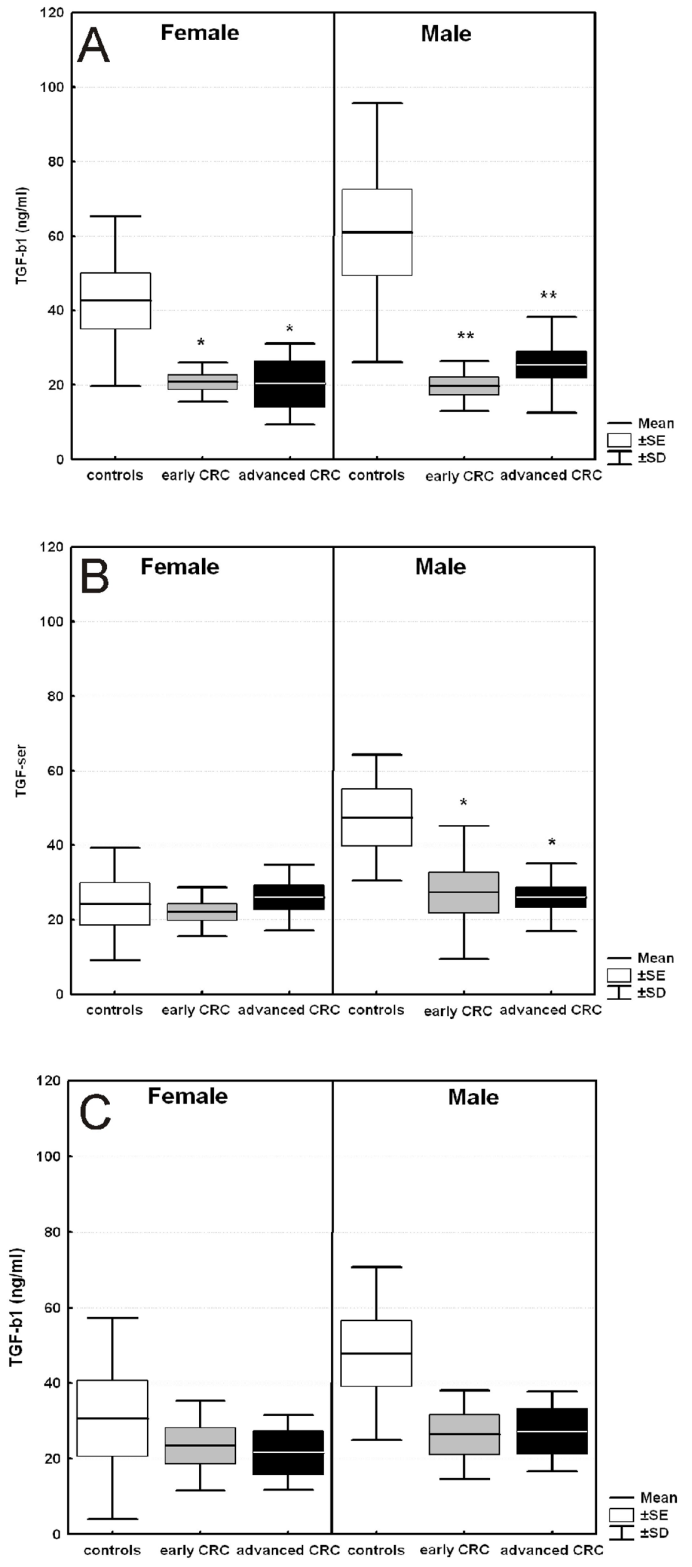
Serum levels of TGF-β1 in cases and controls over the age of 50 yrs. (A) Male and female carriers of *TGFB1* -509CC genotype. (B) Male and female carriers of *TGFB1* -509CT genotype. (C) Male and female carriers of *TGFB1* -509TT genotype. The results are presented as the mean value ±SE (box) and ±SD (whisker). * p-value < 0.05, ** p-value < 0.01.

The serum TGF-β1 levels in patients in early and advanced stages of CRC compared to those of controls stratified by both *TGFB1*–509 C/T polymorphism genotypes and gender are shown in Fig 4.

The reduction in serum TGF-β1 levels is significant in men in early and advanced stages of CRC with at least one C-allele in their *TGFB1*–509 C/T polymorphism genotype (CC and CT); however, this is not the case in women. Significantly lower TGF-β1 levels were found in CRC patients with the CC genotype than in controls with the same genotype, and this result is more prominent in males (Fig 4A). Among men, early and advanced cases showed approximately 3-fold lower serum TGF-β1 levels than controls (19.7±2.4 ng/ml for early; 25.4±3.6 for advanced vs. 60.95±11.6 ng/ml; p<0.01), while in women, the reduction in TGF-β1 was approximately 2-fold (20.7±1.98 ng/ml for early; 20.3±6.2 for advanced vs. 42.5±7.6 ng/ml; p<0.05). In the heterozygous genotype, significantly lower TGF-β1 levels were observed only among male patients in contrast to females (Fig 4B). In males, the reduction in TGF-β1 was significant in early and advanced CRC compared to the controls with the same CT genotype (p<0.05). In contrast, women with early and advanced CRC showed almost the same TGF-β1 levels as controls (p>0.3). Additionally, patients with the TT genotype showed similar or insignificantly decreased TGF-β1 compared to the controls with the same genotype (Fig 4C).

#### Quantification of *TGFB1* mRNA in primary tumor and distant metastasis tissues in patients

**Fig 5:**
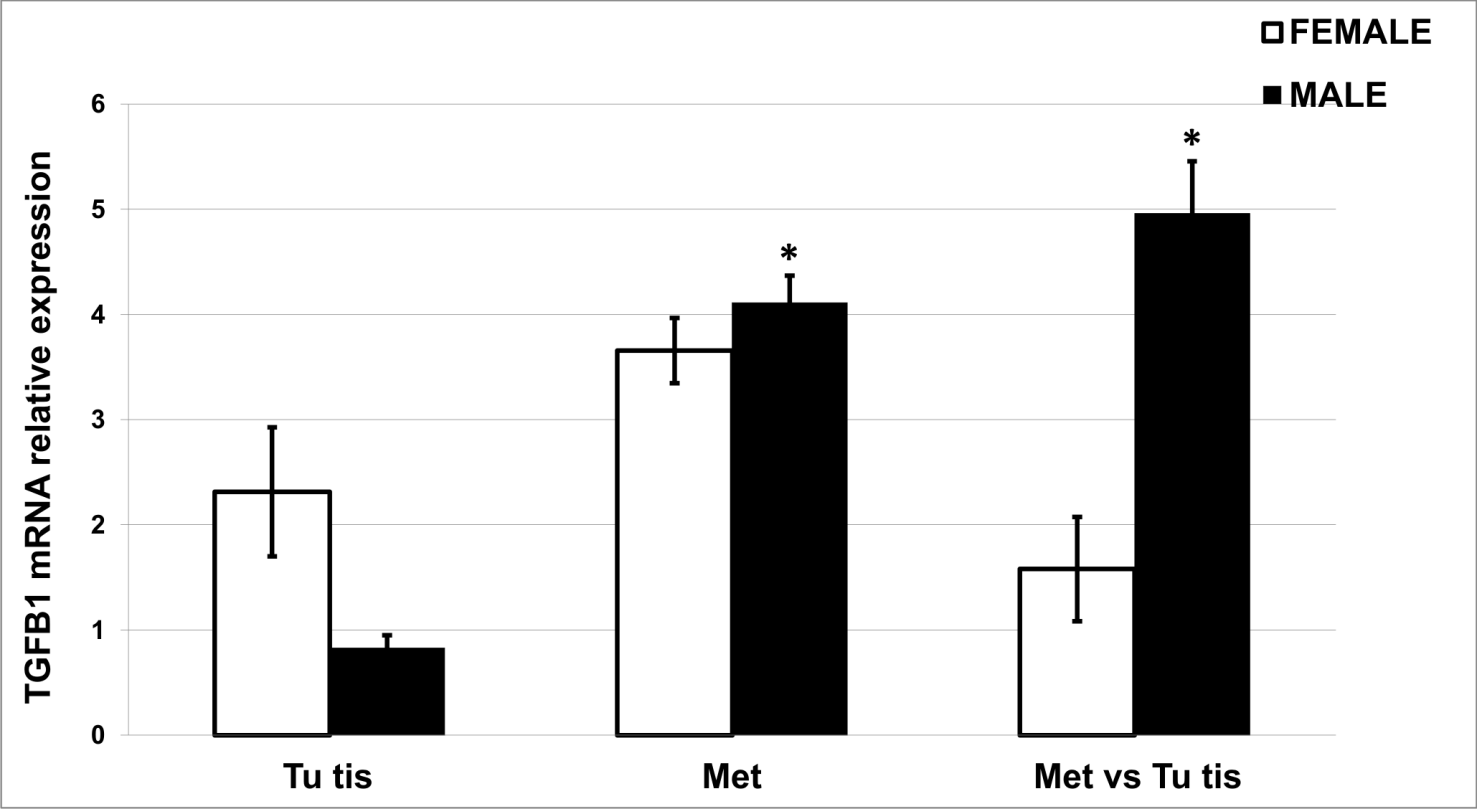
*TGFB1* mRNA relative expression in tumor tissues and distant metastases. mRNA quantification was performed in tissue samples from CRC patients with 4 stage CRC, carriers of *TGFB1* -509CC+CT genotypes in relation to the gender. The relative gene expression was determined by the 2^−ΔΔCt^ method in primary tumor tissue (Tu tis) and in distant metastases (Met) after normalization to the housekeeping reference genes (GAPDH and 18srRNA) and calibrated to the paired non-tumoral mucosa or to the primary tumor tissue (Met vs. Tu tis). Statistical significance is shown compared to the calibrator. * p<0.05.

Quantitative *TGFB1* mRNA expression was determined in paired non-tumoral mucosa, primary tumor and distant metastasis tissues from a relatively small group (n = 14) of CRC patients with TNM stage 4 with at least one C allele in their genotype (CC and CT). Overall, *TGFB1* was significantly upregulated in distant metastases calibrated to both non-tumoral tissues (RQ = 3.655; p = 0.007) and primary tumor tissues (RQ = 2.639; p = 0.026). Sex differences in *TGFB1* mRNA levels are shown in Fig 5.

A twofold increase (RQ = 2.313) in *TGFB1* mRNA was observed in tumor tissues compared to adjacent normal tissues in female patients in contrast to male patients. Upregulated *TGFB1* expression was detected in distal distant metastases compared to non-tumoral mucosa for both males (RQ = 3.655) and females (RQ = 4.112) but only reached significance for male patients (p = 0.035). When *TGFB1* mRNA levels in tumor tissues were compared to those of distant metastases, a significant elevation was observed in males (RQ = 4.959; p = 0.022)

## Discussion

In this case-control study, we examined the distribution of the allele and genotype frequencies of *TGFB1* -509C/T polymorphism in colorectal cancer patients and its association with serum and tumor tissue TGF-β1 expression in a gender-dependent manner. We observed that the development of colorectal neoplasia is associated with diminished serum acid-activated latent TGF-β1 levels in CRC patients compared to healthy controls with homozygous CC genotypes and increased *TGFB1* gene mRNA levels in distant metastases. These data could contribute to the overall assessment of an increased risk for CRC development, especially in patients with at least one C allele in their genotype.

The dual role of TGF-b1 in early and advanced cancer is well known [8,9,10], but we for the first time demonstrate the role of this cytokine depends of gender. The significance of our findings relate to a different role of the studied cytokine and its functional polymorphism in colorectal cancer development in both male and female patients. This might explain at least part of the discrepancies between published data about role of TGF-b1 in colorectal cancer development. TGF-β1 plays important roles in the proliferation and differentiation of the intestinal epithelium and thus in colorectal carcinogenesis. Tumor suppressive functions are mainly manifested by the inhibition of cell proliferation with the simultaneous induction of programmed cell death in early carcinogenesis. In normal epithelial cells, TGF-β1 signalling through the canonical pathway inhibits proliferation by inducing the production of cyclin-dependent kinase inhibitors and repressing the proto-oncogene c-myc [22, 23, 7]. Additionally, the anti-inflammatory activity of this cytokine in the mucosa enhances anti-tumor activities by suppressing tumor-elicited inflammation. Disruption of normal TGF-β1 signalling control is one mechanism for promoting colorectal tumor development and progression, but the factors that influence this process and form a sophisticated system have yet to be completely understood.

The TGF-β1 gene -509C/T promoter polymorphism may directly influence the expression profiles by recruiting transcriptional factors, particularly AP1 [13]. We suppose that this affect strongly depends on the given network between signal transduction pathways in concrete functional cell activity that are determined by specific activation in different diseases. In that way, the variant T allele is associated with increased synthesis of both mRNA and protein in allergy patients [24], though TGF-β1 levels from hepatitis E patients having the CT genotype were significantly higher than those having CC or TT genotypes [25]. Moreover, hepatocellular carcinoma (HCC) patients with the CC genotype had statistically significantly higher plasma TGF-β1 levels and liver tumor tissue TGF-β1 mRNA levels than individuals with the TT genotype [26]. Our study adds new data regarding the effect of the TGF-β1 gene -509C/T promoter polymorphism on serum acid-activated latent TGF-β1 quantities in healthy subjects and patients with colorectal cancer. In this study, we first examined serum TGF-β1 levels in association with the *TGFB1* -509C/T polymorphism in a large group of healthy control subjects. We generally observed significant differences in serum TGF-β1 quantities depending of age and gender in combination with genotype in the healthy control group. Next, we conducted separate analyses on male and female participants depending on their -509C/T *TGFB1* genotype. We detected that TGF-β1 level increased with age in men in the following order: CC>CT>TT. The correlation coefficient for all three genotypes is significant, though it is strongest for the CC genotype (r = 0.62). A similar correlation was observed for the CC genotype in females in contrast to the other two genotypes (CT and TT). Moreover, when serum TGF-β1 levels were compared in males and females above 50 years old, significant differences were calculated only for the CC genotype. Our results suggest that the *TGFB1* -509CC genotype is the highest producing genotype in healthy individuals and that this production is positively correlated with age.

Significantly increased active TGF-β1 plasma levels in elderly individuals aged 86–94 years compared to middle-aged individuals (32–59 years) have been observed previously by Forsey RJ and colleagues [27]. Moreover, the age-related increase in inflammation (“inflamm-aging” or “immunosenescence”) is well documented, as is the increase in many inflammatory cytokines [28, 29]. We suppose that increased TGF-β1 is at least one compensatory mechanism in elderly people for diminishing age-related inflammation. Sex-related differences in serum TGF-β1 levels were observed, with significantly higher levels in men than in women in the healthy control group; these results have also been published previously [30].

When serum TGF-β1 levels from CRC patients were compared to those of controls separated by sex, a significant difference was observed for males only (Fig 3A). We thought that the decrease in the total TGF-β1 was a consequence of colorectal carcinogenesis and that the detected increase in serum TGF-β1 after curative surgery could be a possible prognostic marker. We previously observed significantly increased *TGFB1* and IL-10 gene mRNA levels in peripheral immune cells from CRC patients’ blood before surgery in comparison with healthy donors [31]. This discrepancy could be due to the differential regulation of gene expression and protein secretion caused by both tumors inducing aberrant changes and host anti-tumor responses. It should also be noted that acid-activated latent TGF-β1 has to be activated before binding to TGFBRs.

In this case-control study, a significantly increased risk for advanced CRC was found for the CC genotype. The meta-analysis by Fang and colleagues showed that the *TGFB1* -509C allele is a risk factor for developing colorectal cancer in Asians, though no significant association was found among Europeans [15]. In another meta-analysis, Yang Liu and colleagues suggested that the T-allele of the *TGFB1* -509C/T polymorphism might contribute to a decreased risk in colorectal cancer susceptibility, especially in Caucasians [16].

A recent meta-analysis indicated that the TGF-β1 gene promoter -509C allele might be a risk factor for colorectal cancer based on 4440 patients and 6785 controls [32]. Collectively, all these data and our results raised the possibility that the TGF-β1 –509C allele might contribute to an increased risk for both colorectal cancer susceptibility and progression by stimulating invasion in advanced stages of CRC, which could lead to distant metastases. Moreover, the result of a significantly increased risk for males with CC genotype could perhaps explain at least some discrepancies between previously published data.

In contrast to serum TGF-β1 production and/or activation, tumor tissue expression of *TGFB1* as mRNA or protein was generally associated with tumor progression. Langenskiold and colleagues found that TGF-β1 protein expression in both tumor tissues and plasma was significantly higher in patients with metastatic colorectal cancer than in those without metastatic disease [33]. The correlation of immunohistochemical expression with the clinicopathological parameters associated with CRC revealed that high TGF-β expression and low TGF-βR1, TGF-βR2, Smad4, pSmad2/3, and E-cadherin expression were correlated with the tumor–node–metastasis (TNM) stage of the disease [34]. Finally, the meta-analysis by Chen XL and colleagues in 2017 showed that TGF-β tissue expression can be used as a prognostic biomarker for CRC patients undergoing surgery, especially for CRC patients from Western countries [35].

Recent data suggest that the dual role of TGF-β1 in cancer, also known as the TGF-β1 paradox, may involve the differential intracellular pathway signalling between benign and malignant cells [8]. Another possibility for the dual role of TGF-β might be the distinct roles of circulating- and tissue-expressed TGF-β1 in CRC initiation and progression. While intratumoral TGF-β1 expression strongly promotes carcinogenesis by acting as a major inducer of epithelial to mesenchymal transition (EMT), sustained angiogenesis and evasion of immune surveillance [36], circulating TGF-β1 could exert opposite effects on both epithelial and blood immune cells. Part of this difference could be due to the distinct TGF-β1 influences on target cells by activating discrete signalling pathways that depend on the genetic and epigenetic background of cells. Collectively, TGF-β1 is involved in several intracellular signalling pathways that differ in their consequences, depending on the cell type, grade of differentiation and stage of neoplasm transformation.

We hypothesize that a decrease in serum TGF-β1 drives changes in blood immune cells, which could be a reason for enhanced inflammation, including gastrointestinal inflammation. A seminal paper by Principe and colleagues using genetically modified mice (APC and ATG mutants) demonstrated that systemic TGFBR deficiency leads to changes in cancer-associated cytokines in the serum, including decreased TGF-β1 and IL-10, and a strongly enhanced inflammatory response in the colon, which is predominantly mediated by myeloid cells [37]. Our previous observation of cancer-associated reprogramming of gene expression in blood cells, including *TGFB1* transcriptional alterations, also supports that suggestion [31]. Thus, one mechanism by which circulating TGF-β1 inhibits tumorigenesis may be by suppressing tumors initiating gastrointestinal inflammation. Another possibility for a tumor-suppressing role of circulating TGF-β1 could be the inhibition of IL-6 trans-signalling in colon cancer [38]. Furthermore, in 2012, Lampropoulos and colleagues demonstrated a clear association between cancer-specific overall survival and high TGF-β using Kaplan–Meier survival curves [34].

In conclusion, we present data that systemic TGF-β1 could have a preventive role against colorectal cancer development in a gender-specific manner. The TGF-β1 gene –509C/T promoter polymorphism might contribute to an increased risk of CRC; in particular, males with the CC genotype have a greater risk for CRC development and progression. Colorectal cancer tissue expression of *TGFB1* gene mRNA correlates with tumor progression and metastasis.
